# Implementation of the kidney protection strategy in critically ill patients with acute kidney injury – a multi-center prospective cohort study

**DOI:** 10.1186/s13054-026-06144-0

**Published:** 2026-06-19

**Authors:** Mahan Sadjadi, Matteo Marcello, Andrea Köhler, Fabian Perschinka, Sebastian Schauflinger, Michael Joannidis, István Vadász, Faeq Husain-Syed, Margreet Klop-Riehl, Peter Pickkers, Gianluca Villa, Tobias Nagel, Eike Bormann, Hendrik Booke, Ludwig Maximilian Schöne, Thilo von Groote, Moritz J. Mertes, John A. Kellum, Christian Strauß, Alexander Zarbock

**Affiliations:** 1 Department of Anesthesiology, Intensive Care and Pain Medicine, University Hospital Münster, Albert-Schweitzer-Campus 1, Geb. A1, Münster, 48149 Germany; 2 Department of Nephrology, Dialysis and transplantation, San Bortolo Hospital, Vicenza, Italy; 3 Division of Intensive Care and Emergency Medicine, Department of Internal Medicine, Medical University Innsbruck, Innsbruck, Austria; 4 Department of Internal Medicine II, Justus Liebig University, Universities of Giessen and Marburg Lung Center, Giessen, Germany; 5 Department of Intensive Care, Radboud University Medical Center, Nijmegen, The Netherlands; 6 Department of Anesthesia and Intensive Care, Section of Oncological Anesthesia and Intensive Care, AOU Careggi, Florence, Italy; 7 Institute of Biostatistics and Clinical Research, University Hospital Münster, Münster, Germany; 8 Center for Critical Care Nephrology, Department of Critical Care Medicine, University of Pittsburgh, Pittsburgh, USA

**Keywords:** Acute kidney injury, Critical care, Guideline adherence, Kidney protection strategy, KDIGO, Implementation

## Abstract

**Background:**

The international Kidney Disease: Improving Global Outcomes (KDIGO) guidelines recommend the implementation of a kidney protection strategy (KPS) in patients at high risk of and with Acute Kidney Injury (AKI). However, real-world implementation of this strategy in critically ill patients with AKI is unclear. We quantified timely and sustained adherence to KPS in critically ill adults with moderate-to-severe (KDIGO stage 2 or 3) AKI and explored associations with clinical outcomes.

**Methods:**

This was a multicenter, prospective cohort study enrolling adult patients with moderate or severe AKI requiring vasopressors and/or mechanical ventilation across five centers in Europe. The primary endpoint was adherence to the KPS, which included hemodynamic monitoring, sustained optimization of mean arterial pressure (MAP) > 65 mmHg, monitoring of serum creatinine and urine output, and avoidance of hyperglycemia, radiocontrast agents and nephrotoxins when possible, within 12 h after AKI diagnosis for 48 h or until ICU discharge. Exploratory analyses examined associations between adherence and renal outcomes.

**Results:**

A total of 258 patients were enrolled (median age 69 years [IQR 62–75]; 65% male; median SOFA 10 [IQR 8–13]). The complete KPS was implemented in 80 patients (31%; 95% CI, 25.5–37.2%). Adherence to individual components of the KPS varied widely with optimization of MAP showing the lowest implementation rate (33%). In exploratory analyses accounting for death as a competing risk, KPS adherence was associated with a lower incidence of AKD beyond day 7 (subdistribution hazard ratio [SHR] 0.64; 95% CI, 0.41–0.99; *p* = 0.046), a higher incidence of renal recovery at hospital discharge (SHR 6.02; 95% CI, 4.00–9.05; *p* < 0.0001), and a lower incidence of RRT within 30 days (SHR 0.12; 95% CI, 0.02–0.91; *p* = 0.04). After multivariable adjustment, the association with renal recovery remained robust (adjusted SHR 6.29; 95% CI, 3.08–12.85; *p* < 0.0001). A clear dose-response relationship was observed between the number of implemented KPS components and renal outcomes.

**Conclusions:**

In critically ill patients with moderate-to-severe AKI, the complete KDIGO-recommended kidney protection strategy was implemented in approximately one-third of patients, and full KPS adherence was associated with a higher rate of renal recovery at hospital discharge.

**Supplementary Information:**

The online version contains supplementary material available at 10.1186/s13054-026-06144-0.

## Highlights

In this multicenter prospective cohort study of 258 critically ill adults with moderate or severe AKI, the complete KDIGO-recommended kidney protection strategy (KPS) was implemented in 31% of patients.

Sustained optimization of mean arterial pressure (33%) and hyperglycemia management (43%) were the most significant care gaps.

In competing-risk regression accounting for death, KPS adherence was independently associated with a lower rate of AKD beyond day 7, a higher rate of renal recovery at hospital discharge, and a lower incidence of RRT within 30 days.

The association between number of implemented KPS components and renal outcomes displayed a dose-response relationship with each implemented component being associated with lower risk of adverse renal outcomes.

**Supplementary Information:**

The online version contains supplementary material available at 10.1186/s13054-026-06144-0.

## Introduction

Acute kidney injury (AKI) is a global problem affecting more than 10% of all hospitalized patients and up to 50% of critically ill patients, with severity and duration of AKI affecting morbidity and mortality [1, 2]. Evidence demonstrates that two-thirds of patients with AKI resolve their renal dysfunction within three to seven days whereas those in whom renal dysfunctions persist have dramatically reduced survival [2]. Persistence of AKI is of importance as it increases the risk of developing chronic kidney disease (CKD) which is a major cause of morbidity and long-term mortality. This link between AKI and CKD has been established over the last decade [3]. To date, there are no specific pharmacological options for preventing or treating AKI, which is why implementation of existing secondary preventive measures is paramount to reduce its occurrence and mitigate its consequences.

The Kidney Disease: Improving Global Outcomes (KDIGO) clinical practice guidelines describe a kidney protection strategy (KPS) which consists of different supportive measures, including discontinuation of nephrotoxic agents when possible, functional hemodynamic monitoring, optimization of volume status and mean arterial pressure, monitoring of serum creatinine and urine output, avoidance of hyperglycemia, and avoidance of radiocontrast agents – which should be initiated in all patients at risk of or with AKI [4]. Several randomized trials demonstrated that the implementation of the kidney protection strategy (KPS) in patients at high risk of AKI identified by biomarkers significantly reduced the occurrence of moderate/severe AKI after major surgery [5–10]. However, despite this evidence, the implementation of the KDIGO-recommended KPS in patients at high risk of AKI is still not standard clinical practice [11]. The extent to which the KPS is applied to patients with established AKI and whether the strategy positively influences the trajectory of AKI is unknown. This multicenter, multinational observational study therefore aimed to determine the proportion of patients with moderate or severe AKI receiving the complete KDIGO-recommended kidney protection strategy, to quantify the extent to which each component is implemented, and to explore associations between KPS adherence and renal outcomes.

## Methods

### Study design and setting

This was a prospective multicenter cohort study conducted across five hospitals in Europe (Academic centers in Münster and Gießen [Germany], Innsbruck [Austria], Nijmegen [The Netherlands] and Florence [Italy]). The primary ethics approval was obtained from the Research Ethics Committee of the Chamber of Physicians, Westfalen-Lippe and the University of Münster (2023-708-f-S), and each site sought local approval from the responsible committee. While the study was not preregistered in a public registry, the full study protocol, including all predefined primary and exploratory endpoints as well as the statistical analysis plan were approved by the ethics committee prior to enrollment of the first patient. Written informed consent was obtained from all participants or their legal representatives per local regulations and the ethics approval. Reporting follows the Strengthening the Reporting of Observational Studies in Epidemiology (STROBE) guidelines [12].

### Participants

Eligible patients were critically ill adults (≥ 18 years) with moderate or severe AKI (KDIGO stage 2 or 3) who required vasopressors and/or mechanical ventilation at the time of enrollment. Patients were eligible at any point during their ICU stay upon first meeting these criteria. Patients were excluded from the study if they had any of the following exclusion criteria: CKD with a glomerular filtration rate < 20 ml/min/1.73m^2^, chronic dialysis dependency, history of renal transplantation, permanent ligation of the renal arteries, AKI immediately following nephrectomy, or dependency on the investigator or employment by the sponsor or investigator.

### Procedures and measurements

Following AKI onset, with a pre-specified 12-hour grace period for KPS implementation, each component of the KPS was assessed continuously by trained study personnel (physicians and research coordinators) at each site using standardized case report forms for a period of 48 h or until the patient was discharged from the ICU. Data were verified retrospectively against electronic health records by a physician who was not involved in the study procedures. The KDIGO guideline-recommended KPS was operationalized into seven discrete measurable components as follows:

1. Avoidance of nephrotoxic agents when possible – measured as whether administration of an agent was deemed absolutely necessary (e.g., antibiotics administered according to results of microbiological testing). In the case of a lack of suitable alternatives, this was not considered a violation of KPS adherence. This clinical judgment was performed and documented by the treating physician in the case report form and subsequently verified by the site investigator.
2. Regular functional hemodynamic monitoring – defined as assessment of the hemodynamic status using echocardiography, transpulmonary thermodilution (e.g., PiCCO) or related methods at least three times daily for 48 h or until ICU discharge. The mode of hemodynamic monitoring was at the discretion of the treating physician.
3. Sustained optimization of mean arterial pressure (MAP) – defined as avoidance of MAP < 65 mmHg. For patients with continuous invasive arterial pressure monitoring, MAP optimization was assessed by reviewing the continuous recording for episodes of MAP < 65 mmHg lasting longer than 5 min. For patients without continuous monitoring, a violation was defined as two consecutive MAP measurements < 65 mmHg.
4. Close monitoring of serum creatinine – daily serum creatinine measurement.
5. Close monitoring of urine output – hourly urine output documentation. A breach was defined as a gap in hourly documentation exceeding one consecutive hour during the 48-hour observation window.
6. Avoidance of hyperglycemia – defined as avoidance of blood glucose > 180 mg/dL for longer than two consecutive measurements without corrective measures (e.g., initiation of insulin therapy).
7. Avoidance of intravenous radiocontrast agents – defined as no administration of radiocontrast unless absolutely necessary for timely diagnosis of a life-threatening condition. The necessity of contrast administration was adjudicated by the treating team and subsequently verified by the site investigator.

### Outcome measures

The primary outcome was adherence to all components of the KDIGO-recommended KPS within 12 h of moderate/severe AKI diagnosis and sustaining the components for 48 h or until ICU discharge – whichever occurred first. Secondary outcomes were adherence rates to each of the KPS components. Exploratory outcomes were renal recovery at hospital discharge (defined as absence of AKI criteria and an eGFR decline < 25% from baseline eGFR), persistence of AKD (defined as AKI or GFR < 60 ml/min/1.73m^2^, or decrease in GFR by ≥ 35% times baseline, or increase in SCr by ≥ 50% times baseline for at least 7 days or more), as well as any use of renal replacement therapy (RRT), and their associations with KPS implementation.

### Power calculation

Based on preliminary data suggesting an expected bundle adherence rate of approximately 20%, the sample size was calculated to ensure that the exact (Clopper-Pearson) 95% confidence interval for the primary endpoint would have a width not exceeding 10% (margin of error ± 5%), requiring 246 evaluable patients. Assuming a dropout rate of 5%, 258 patients were to be recruited. The sample size was calculated using R (Version 4.3.1).

### Statistical analysis

The study population is described using summary statistics: mean ± standard deviation (SD) for normally distributed continuous variables, median and interquartile range (IQR) for skewed continuous variables, and frequencies for categorical variables. Differences in baseline characteristics were explored between patients in whom the KPS was implemented successfully and those in whom this was not achieved using chi-square or Fisher’s exact tests for categorical variables and the Wilcoxon rank-sum test for continuous variables.

For the primary analysis, the proportion of patients receiving the complete KPS within 12 h of moderate/severe AKI and sustaining the components for 48 h or until ICU discharge was estimated with an exact 95% confidence interval (Clopper-Pearson method). Partial strategy adherence was characterized by reporting the distribution of the number of components fulfilled (0–7).

All analyses investigating associations between KPS implementation and clinical outcomes were considered exploratory, given the observational design and the absence of a formal power calculation for these comparisons. In this critically ill population with stage 2–3 AKI, in-hospital mortality was substantial and differed markedly between groups, creating a significant competing risk for all renal outcomes: patients who die before assessment of renal endpoints is possible cannot contribute events, systematically distorting standard regression comparisons. Accordingly, all associations between KPS adherence and renal outcomes (AKD at day 7, renal recovery at hospital discharge, and renal replacement therapy at 30 and 90 days) were analyzed using Fine-Gray subdistribution hazard regression, with death treated as a competing event according to the appropriate timeframe by variable. For each renal endpoint, we report the subdistribution hazard ratio (SHR) with 95% confidence intervals from both univariable and multivariable models. Multivariable models included prespecified covariates selected on the basis of clinical relevance and prior literature. In exploratory analyses of a potential dose-response relationship, we first fitted unadjusted models including the number of KPS components fulfilled as a continuous predictor for each renal endpoint. To explore potential nonlinearity in the relationship, we fitted restricted cubic spline models with three degrees of freedom. These models were then adjusted for prespecified covariates (age, baseline AKI stage, SOFA score, APACHE II score, and history of CKD). Global nonlinearity was assessed using Wald tests on the spline terms. Subdistribution hazard ratios (SHR) with 95% confidence intervals were reported. To characterize the distribution of non-fulfillment, we computed pairwise phi coefficients between all KPS component violations (Supplementary Fig. 2). As a sensitivity analysis, we estimated a propensity score for complete KPS adherence using logistic regression including all baseline characteristics listed in Table 1. Inverse probability of treatment weighting (IPTW) was applied to Fine–Gray subdistribution hazard models for the key renal outcomes (Supplementary Table 4, Supplementary Fig. 4). Multivariable models were additionally adjusted for diabetes mellitus and hypertension, given their known associations with renal outcomes and their differential distribution between groups (Supplementary Table 5). An exploratory SHR analysis was also done for individual KPS component effects (Supplementary Table 3). All analyses were conducted using a complete-case approach; no imputation of missing values was performed. Statistical analyses were performed using R version 4.4.3.

**Table 1:** Baseline characteristics and comorbidities (*N* = 258)

Characteristic	All patients (*N* = 258)	KPS fulfilled (*n* = 80)	KPS Not fulfilled (*n* = 178)	*p*
**Demographics**				
Age, years – median [IQR]	69 [62–75]	70 [65–75]	68 [62–76]	0.206
Male sex – n (%)	167 (65.0)	49 (61.3)	118 (66.3)	0.396
Height, cm – mean ± SD	173.6 ± 10.2	172.6 ± 9.8	174.1 ± 10.4	0.253
Weight, kg – median [IQR]	82 [70–95]	80 [68–96]	84 [70–93]	0.729
**Illness severity**				
SOFA score – median [IQR]	10 [8–13]	11 [9–14]	10 [8–12]	0.068
APACHE II – median [IQR]	24 [19–29]	25 [19–32]	24 [20–29]	0.541
**Comorbidities – n (%)**				
Hypertension	151 (58.5)	34 (42.5)	117 (65.7)	0.001
Coronary artery disease	65 (25.2)	17 (21.3)	48 (27.1)	0.43
Heart failure (NYHA ≥ I)	39 (15.4)	10 (12.5)	29 (16.4)	0.74
Diabetes mellitus	65 (25.2)	19 (23.8)	46 (26.0)	0.72
Peripheral vascular disease	27 (10.6)	7 (8.8)	20 (11.3)	0.66
COPD	32 (12.6)	12 (15.0)	20 (11.3)	0.41
Chronic liver disease	37 (14.5)	14 (17.5)	23 (13.0)	0.34
Malignancy	54 (21.1)	23 (28.8)	31 (17.5)	0.05
History of myocardial infarction	23 (9.0)	4 (5.0)	19 (10.7)	0.16
History of stroke	24 (9.4)	9 (11.3)	15 (8.5)	0.49
Dementia	4 (1.6)	1 (1.3)	3 (1.7)	1.00
**ASA physical status – n (%)** ^a^				0.27
I II III IV	27 (10.5) 87 (33.7) 88 (34.1) 55 (21.3)	7 (8.6) 22 (27.2) 31 (38.3) 20 (25.0)	20 (11.3) 65 (36.7) 57 (32.2) 35 (19.8)	
**Ventilatory support – n (%)** ^a^				0.31
No mechanical ventilation	65 (25.3)	19 (23.8)	46 (26.0)	
Non-invasive ventilation	26 (10.1)	5 (6.3)	21 (11.9)	
Invasive mechanical ventilation	166 (64.6)	56 (70.0)	110 (62.1)	
**Renal parameters**				
Creatinine at hospital admission, mg/dL – median [IQR]	1.4 [0.91–1.98]	1.4 [1.0–1.9]	1.33 [0.9–2.0]	0.93
Creatinine at ICU admission, mg/dL – median [IQR]	1.5 [1.0–2.3]	1.45 [1.0–2.3]	1.5 [1.0–2.3]	0.81
Baseline eGFR, mL/min/1.73 m² – median [IQR]	68 [56–90]	70 [54–90]	66 [56–90]	0.97

Data are presented as median [interquartile range], mean ± standard deviation, or n (%). ^a^*n*=257. Abbreviations: APACHE, Acute Physiology and Chronic Health Evaluation; ASA, American Society of Anesthesiologists; COPD, chronic obstructive pulmonary disease; eGFR, estimated glomerular filtration rate; IQR, interquartile range; KPS, Kidney Protection Strategy; NYHA, New York Heart Association; SD, standard deviation; SOFA, Sequential Organ Failure Assessment

## Results

**Fig. 1:**
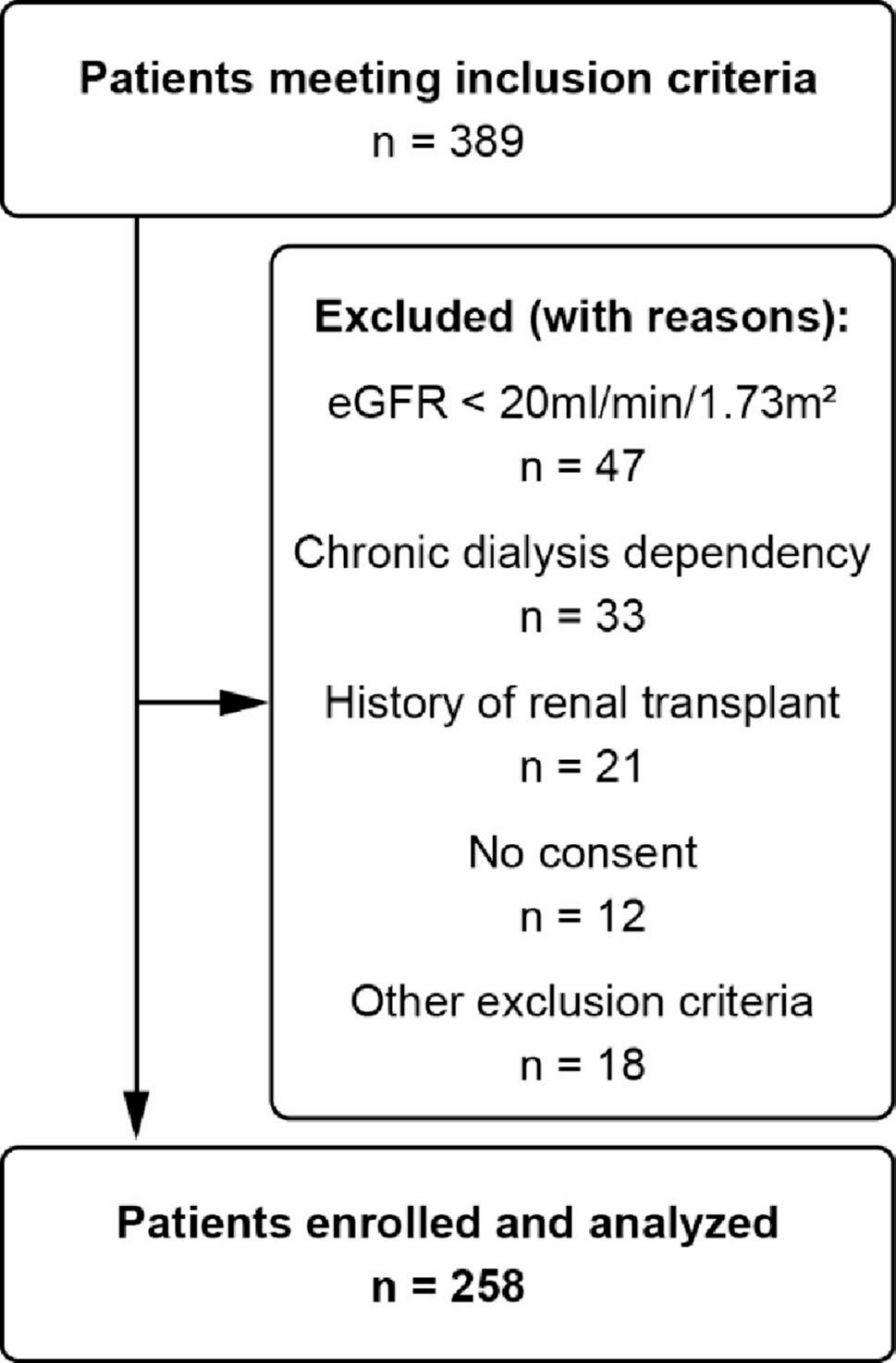
Study flowchart

Between July 2024 and December 2025, 258 patients were enrolled across five European centers and included in the analysis (Fig. 1, Study Flowchart). Baseline characteristics are summarized in Table 1. The median age was 69 years (IQR 62–75), and 65% were male. Illness severity at ICU admission was high, with a median SOFA score of 10 (IQR 8–13) and a median APACHE II score of 24 (IQR 19–29). At ICU admission, 166 patients (65%) required invasive mechanical ventilation, and the median SOFA cardiovascular sub-score was 4 (IQR 3–4). The most prevalent comorbidities were hypertension (59%), coronary artery disease (26%), and diabetes mellitus (25%). The median baseline (pre-AKI) estimated GFR was 68 ml/min/1.73 m² (IQR 56–90).

### Primary endpoint: Adherence to the kidney protection strategy

**Fig. 2:**
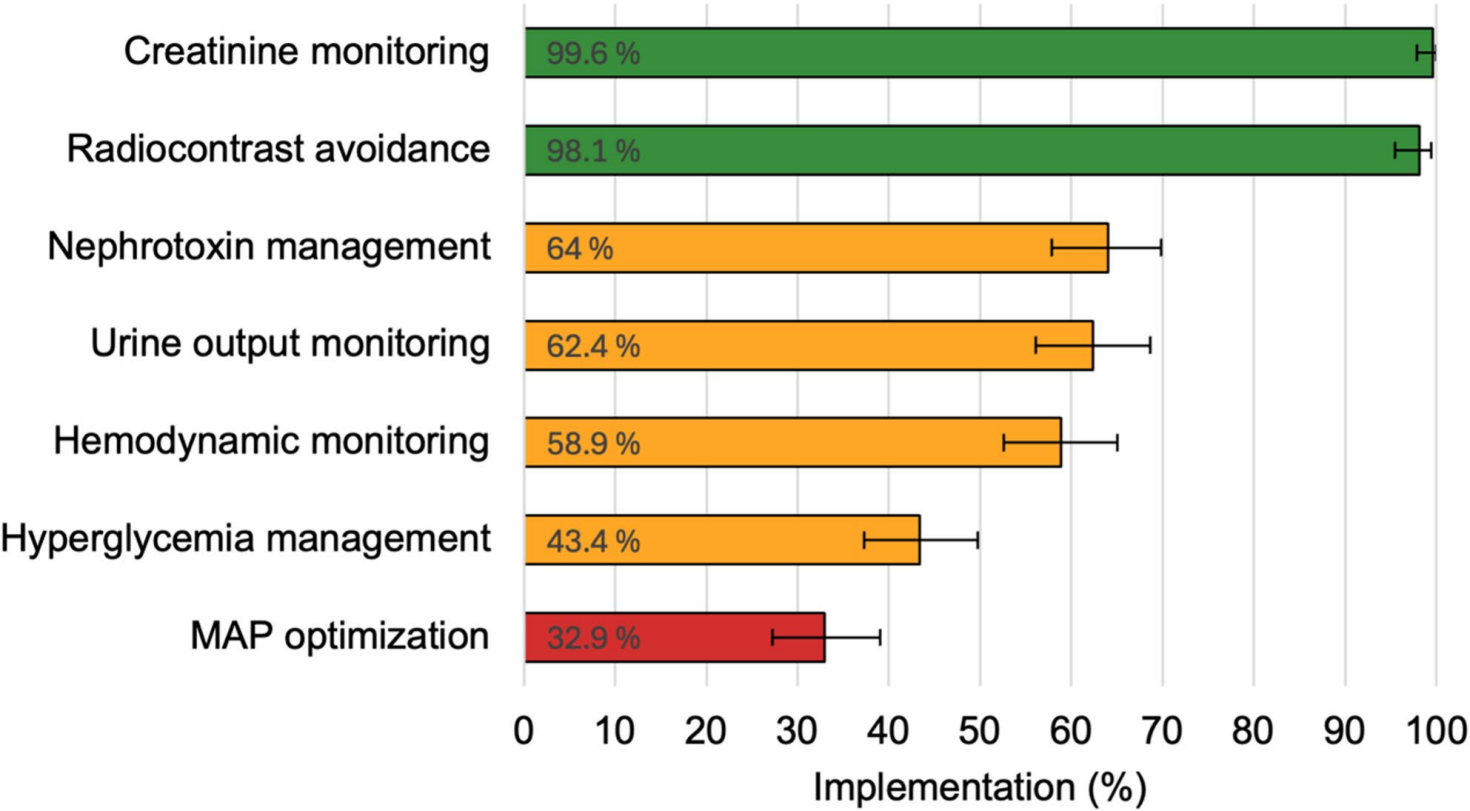
Implementation rates for components of the kidney protection strategy

Complete adherence to the KPS was fulfilled in 80 of 258 patients (31%; 95% CI, 26–37%). The distribution of partial KPS adherence is shown in Supplementary Table 2. Adherence to individual components varied widely (Fig. 2, Table 2). Components with near-universal adherence included daily creatinine monitoring (99.6%) and radiocontrast avoidance (98%). Components with intermediate adherence included avoidance of the use of nephrotoxins (64%), urine output monitoring (62%), and hemodynamic monitoring (59%). The lowest implementation rate was observed for hyperglycemia management (43%) and optimization of MAP (33%).

**Table 2:** Adherence to KPS components within 48 h of AKI onset

KPS component	Adherence, *n* (%)	95% CI for Adherence
Creatinine monitoring	257 (99.6)	97.9–100
Radiocontrast avoidance	253 (98.1)	95.5–99.4
Nephrotoxin management	165 (64.0)	57.8–69.8
Urine output monitoring	161 (62.4)	56.2–68.3
Hemodynamic monitoring	152 (58.9)	52.6–65.0
Hyperglycemia management	112 (43.4)	37.3–49.7
Optimization of MAP	85 (32.9)	27.2–39.0
**Complete KPS (all 7 components)**	**80 (31.0)**	**25.5–37.2**

Adherence defined as sustained compliance with each component for 48 h following AKI onset, with initiation within 12 h of AKI diagnosis. CIs calculated using the Clopper-Pearson exact method. Denominators vary owing to component-specific evaluability. Complete KPS adherence is presented in bold. KPS, kidney protective strategy

### Between-center variation

While the between-center variation in complete KPS adherence was relatively narrow (ranging from 26% to 35%) when excluding the small center 2 cohort, component-level variation was substantially greater. (Supplementary Table 1). The components with the greatest between-center variation were urine output monitoring, with violations ranging from 2% (1/47) at center 1 to 72% (18/25) at center 3, and MAP optimization, ranging from 20% (2/10) at center 2 to 72% (34/47) at center 1. Hyperglycemia management violations ranged from 20% (2/10) to 68% (17/25). This component-level variation suggests that organizational factors influence implementation of specific elements, even if overall bundle completion rates were relatively homogeneous.”

### Clinical outcomes

AKI persisted beyond 48 h in 70% of patients (95% CI, 63–77%) and AKD beyond day 7 was present in 59% of patients (95% CI, 51–67%). Median ICU length of stay was 11.69 days (IQR 6.4–20.9). Renal replacement therapy was required at any time during the ICU stay in 30.5% of evaluable patients (95% CI 24.8–36.7%). Renal recovery at hospital discharge was documented in 58% (95% CI, 49–66%). Renal replacement therapy was required and ongoing in 13% of patients at 30 days and in 7% at 90 days. Thirty-day mortality was 16% and 90-day mortality was 28%.

### Association between kidney protection strategy adherence and outcomes

In univariable competing-risk analyses, KPS adherence was associated with a significantly lower occurrence of AKD beyond day 7 (SHR 0.64; 95% CI, 0.41–0.99; *p* = 0.046), a markedly higher occurrence of renal recovery at hospital discharge (SHR 6.02; 95% CI, 4.00–9.05; *p* < 0.0001), and a lower incidence of RRT within 30 days (SHR 0.12; 95% CI, 0.02–0.91; *p* = 0.040). RRT within 90 days showed a directionally consistent association with KPS adherence (SHR 0.32; 95% CI, 0.04–2.53; *p* = 0.28) (Table 3).

**Table 3:** Association between KPS implementation and renal outcomes

Outcome	Univariable SHR (95% CI)	*p*	Adjusted SHR (95% CI)	*p*
Persistent AKI	0.95 (0.73–1.25)	0.73	0.93 (0.70–1.34)ᵃ	0.82
AKD (> 7 days)	0.64 (0.41–0.99)	0.046	0.71 (0.42–1.20)ᵃ	0.19
Renal recovery	6.02 (4.00–9.05)	< 0.0001	6.29 (3.08–12.85)^b^	< 0.0001
RRT (30 days)	0.12 (0.02–0.91)	0.04	0.16 (0.02–1.11)^b^	0.064
RRT (90 days)	0.32 (0.04–2.53)	0.28	—^c^	—

SHR = subdistribution hazard ratio. An SHR < 1 indicates a lower cumulative incidence of the outcome in the KPS-fulfilled group; an SHR > 1 indicates a higher cumulative incidence. ^a^adjusted for age, AKI stage, SOFA and APACHE-II scores; ^b^adjusted for age, AKI stage, CKD, SOFA and APACHE-II scores; ^c^adjusted model not performed due to limited events. AKD, acute kidney disease; KPS, kidney protection strategy; SHR, subdistribution hazard ratio; RRT, renal replacement therapy

**Fig. 3:**
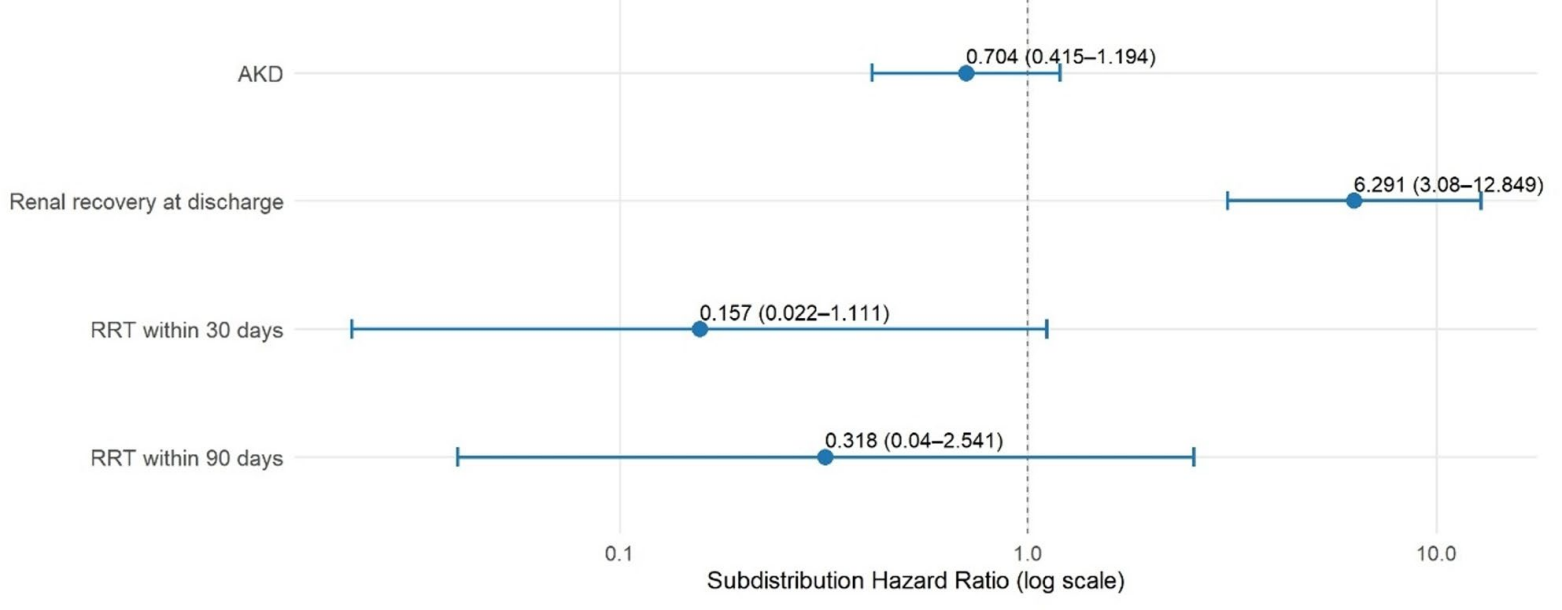
Association between KPS implementation and key renal outcomes. Forest plot showing adjusted subdistribution hazard ratios (SHR) and 95% confidence intervals from multivariable Fine–Gray competing-risk regression models, with death treated as a competing event. An SHR < 1 indicates a lower cumulative incidence of the outcome in the KPS-fulfilled group; an SHR > 1 indicates a higher cumulative incidence. Covariates for AKD: age, AKI stage, SOFA score, and APACHE II score. Covariates for renal recovery and RRT at 30 days: age, AKI stage, CKD, SOFA score, and APACHE II score. RRT at 90 days: unadjusted model only (adjusted model not performed due to limited events)

After multivariable adjustment for prespecified covariates, the association between KPS adherence and renal recovery at hospital discharge remained robust (adjusted SHR 6.29; 95% CI, 3.08–12.85; *p* < 0.0001). The associations with AKD (adjusted SHR 0.71; 95% CI, 0.42–1.20; *p* = 0.19) and RRT within 30 days (adjusted SHR 0.16; 95% CI, 0.02–1.11; *p* = 0.064) were directionally consistent with benefit, but no longer statistically significant after adjustment. Results of all competing-risk analyses are reported in Table 3; Fig. 3.

Other renal outcomes did not differ significantly between groups, but all showed directional associations consistent with a nephroprotective effect (Table 3). All analyses investigating associations between KPS implementation and outcomes are exploratory.

### Exploration of a dose-response relationship

**Fig. 4:**
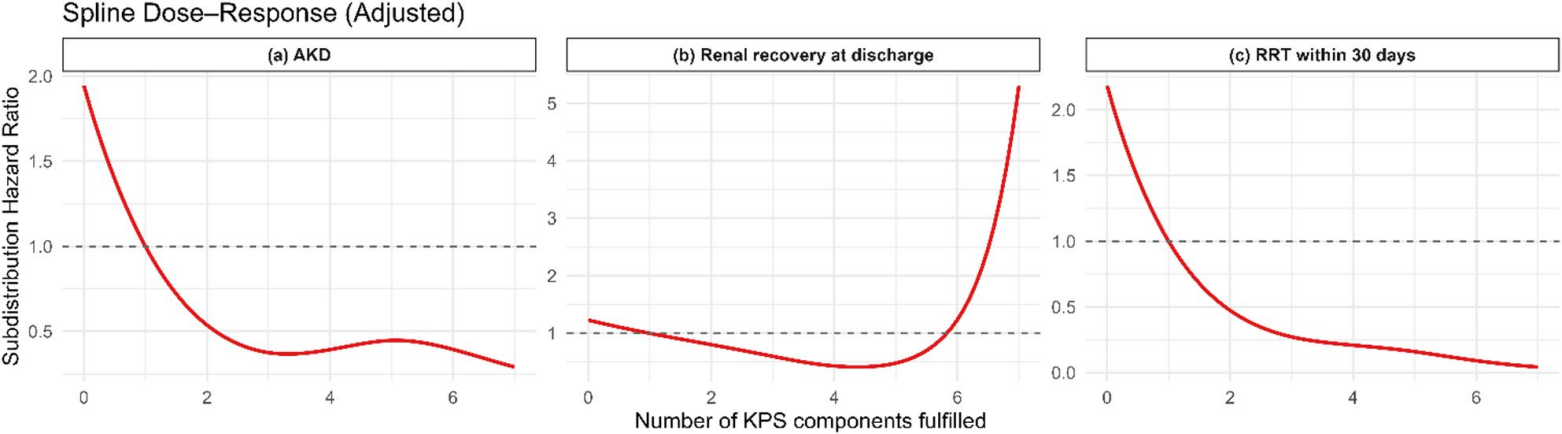
Dose-response relationship between KPS implementation and renal outcomes. Dose-response relationships per additional KPS component implemented were analyzed in spline Fine-Gray models. Analyses are adjusted for age, baseline AKI stage, SOFA score, APACHE II score, and history of chronic kidney disease. Global Wald test for non-linearity: AKD (χ² = 5.14, df = 3, *p* = 0.16), renal recovery (χ² = 19.6, df = 3, *p* = 0.0002), RRT at 30 days (χ² = 10.6, df = 3, *p* = 0.014). Abbreviations: AKD, acute kidney disease; KPS, kidney protection strategy; RRT, renal replacement therapy

In linear Fine-Gray models, each additional KPS component fulfilled was associated with a lower cumulative incidence of RRT within 30 days (unadjusted SHR 0.74; 95% CI 0.57–0.95) and AKD (unadjusted SHR 0.90; 95% CI 0.80–0.98). After adjustment, these associations remained formally significant (RRT within 30 days: adjusted SHR 0.75; 95% CI 0.59–0.97; AKD: adjusted SHR 0.88; 95% CI 0.77–0.99). No significant linear association was observed for renal recovery. Beyond these linear associations, spline models revealed a significant nonlinear association for renal recovery (*p* < 0.001) and RRT within 30 days (*p* = 0.014). The spline curves demonstrated that the relationship between KPS adherence and renal outcomes is not strictly linear. These analyses are exploratory. Spline curves are presented in Fig. 4.

## Discussion

In this prospective multicenter cohort study of 258 critically ill adults with KDIGO stage 2–3 AKI across five European centers, we found that adherence to recommended protective measures of the KPS was profoundly inconsistent and that the complete KPS was implemented in less than one-third of patients. While components embedded in routine ICU workflows such as daily creatinine monitoring were fulfilled in virtually all patients, critical elements like hourly documentation of urine output, hemodynamic monitoring, discontinuation of nephrotoxins when possible, and sustained optimization of MAP were not reliably implemented in daily practice. In exploratory analyses, KPS adherence was independently associated with renal recovery at hospital discharge as well as directionally consistent improvements in all measured renal outcomes.

### Relationship to prior work

Earlier studies have demonstrated that implementation of the KDIGO-recommended KPS as a preventive measure can reduce the incidence and severity of AKI in high-risk populations. The PrevAKI trials demonstrated that biomarker-guided implementation of nephroprotective measures in cardiac surgery patients significantly reduced the occurrence of postoperative AKI [8, 9], and the recently published BigpAK2 trial definitively confirmed this finding in a broad surgical population [10]. However, these trials assessed KPS implementation in controlled, interventional settings with dedicated study personnel ensuring protocol adherence, and they focused on AKI prevention in high-risk patients without established AKI. The low rate of complete KPS adherence observed in our study despite a liberal definition of adherence (e.g. limited only to the immediate ICU course, and with a 12-hour grace period between AKI onset and assessment of KPS implementation) is striking but consistent with broader evidence that multifaceted care strategies in critical care are difficult to fully implement. For example, complete adherence to the Surviving Sepsis Campaign hour-1 bundle has been consistently poor, even under mandates in well-resourced settings [13, 14]. Our findings suggest that the KDIGO-recommended KPS presents a similarly significant implementation challenge, and opportunity for improvement of care.

Our findings extend the available literature in two important ways. First, we document real-world uptake of nephroprotective measures in patients with established moderate-to-severe AKI, demonstrating that at least two-thirds of patients do not receive the complete recommended strategy in a timely manner. Second, we provide exploratory evidence that KPS adherence is associated with improved renal outcomes – specifically, improved renal recovery, with clear indication for a potential dose-response relationship across a range of renal outcomes – suggesting that the KDIGO-recommended KPS may not only prevent AKI but also mitigate its progression once established. This is particularly relevant, as non-reversal of acute injury represents a critical transition point toward CKD [2]. These findings, while exploratory, also help address the relationship between KPS and renal outcomes such as use of RRT which are too uncommon to be evaluated in prevention studies.

### Interpretation and biological plausibility

The 31% complete KPS adherence observed in our study should be interpreted in the context of achievability. Even the BigpAK-2 trial [10], conducted with dedicated study personnel, achieved complete adherence to a similar nephroprotective bundle in only approximately half of intervention-group patients. This comparison suggests that 100% KPS adherence may not be a realistic target and that identifying a reasonable, achievable target – potentially focused on the components most amenable to implementation and most strongly associated with outcomes – represents an important area for future research. The wide variation in component-level adherence follows a predictable pattern. Components that are largely passive, binary, or embedded in routine ICU workflows – daily creatinine measurement, radiocontrast avoidance – were achieved in > 98% of patients. Components requiring active, repeated clinical action – optimization of MAP and intensified hemodynamic monitoring – had low adherence rates around or below 50%. This gradient suggests that the barriers to KPS adherence are not primarily related to awareness of AKI guidelines but rather to the operational complexity of implementing intensive monitoring and treatment targets in the context of competing clinical demands. While the observational design precludes any causal inference, the dose-response relationship observed in the exploratory analyses, and its directional persistence after multivariable adjustment support the biological coherence of a cumulative-benefit framework. It is of note that the dose-response analysis treats all seven KPS components as interchangeable units, which is an important simplification. In reality, the components differ in their nature: some are processes of care largely within the ICU team’s control (e.g., performing hemodynamic monitoring, documenting urine output, ordering daily creatinine measurements), while others reflect the outcome of the interaction between care delivery and the underlying disease process (e.g., maintaining MAP ≥ 65 mmHg). This heterogeneity means that the observed dose-response gradient should be interpreted cautiously: it may partly reflect a gradient in disease severity rather than a purely causal relationship between the number of implemented measures and outcomes.

The observed proportion of 31% KPS adherence was higher than expected, though the precision of the prevalence estimate remains clinically meaningful. By design, our study is at risk of a Hawthorne effect as well as reporting bias due to the social desirability of adhering to recommended guidelines. It may therefore be plausible that the true rate of KPS adherence is even lower.

The association between the implementation of the KPS with the improved rate of renal recovery was robust after multivariate adjustment, and further key renal endpoints were directionally consistent in adjusted analyses. Together, these convergent findings indicate a coherent and clinically meaningful association. While it should be interpreted cautiously, the dose-response finding deserves emphasis as it may suggest that incremental improvements in KPS adherence – even adding one additional component to routine care – could meaningfully reduce the burden of renal impairment and ultimately improve patient-centered outcomes.

These exploratory findings are biologically plausible: the components of the KPS address specific mechanisms of ongoing kidney injury. Discontinuation of nephrotoxins reduces direct tubular insult [15], hemodynamic monitoring enables targeted optimization of renal perfusion [16], MAP optimization maintains renal blood flow above autoregulatory thresholds [17, 18], creatinine and urine output monitoring enable early detection of AKI progression and in the case of urine output, strict monitoring is associated with improved survival [19, 20], hyperglycemia management may reduce oxidative stress and inflammation [21, 22], and radiocontrast media avoidance may prevent additional tubular injury [23]. The concurrent delivery of all components may create a permissive milieu for renal recovery.

### Clinical implications

Despite the observational nature of this study, our findings have several important implications for clinical practice and future research.

First, implementation of recommended protective measures is by no means automatic. The substantial between-center variation observed across virtually all components demonstrates that high adherence to individual elements is achievable in some settings, suggesting that organizational and cultural factors, rather than intrinsic clinical barriers, drive substantial shares of the variation. Sharing best practices across institutions through collaborative quality-improvement networks could narrow these gaps. Second, the identification of specific components with low adherence, particularly the sustained optimization of hemodynamics and MAP, provides actionable targets for quality improvement. Standardized protocols, electronic health record-based alerts for hemodynamic instability, hyperglycemia and nephrotoxin exposure, and structured twice- or thrice-daily checklists could improve uptake of these measures. Third, the low rate of complete KPS adherence challenges the feasibility of implementing measures in all patients and suggests a critical need for a tiered approach or an improvement in our tools to identify patients who stand to benefit most from the implementation of time-consuming components of the KPS. Future research should further determine whether specific components or component combinations drive the association with outcomes, which would allow evidence-based prioritization of implementation efforts. Fourth, these data provide robust baseline estimates of current practice patterns that can serve as benchmarks for quality-improvement programs and as the basis for planning pragmatic implementation trials.

### Limitations

Several limitations warrant caution. First, this is an observational study, and the exploratory associations between adherence and renal outcomes are susceptible to confounding by indication and severity. KPS adherence was assessed as a binary metric for each component (target met or not met), without systematically capturing the clinical context of deviations or the corrective actions taken in response. For example, a transient MAP < 65 mmHg episode that is promptly corrected is clinically distinct from sustained hypotension due to refractory septic shock, yet both would be classified as violations in our framework. This lack of granularity means that some ‘violations’ may reflect appropriate clinical management of unavoidable physiological perturbations rather than true care gaps. Although we adjusted for illness severity in multivariable models, residual confounding cannot be excluded. SOFA and APACHE II scores were captured at ICU admission and may not fully reflect illness severity at the time of AKI onset in patients who developed AKI later during the ICU stay. This temporal gap could introduce residual confounding in the exploratory outcome analyses. Second, even if baseline characteristics were generally similar between groups, some minor differences existed, and while the multivariable models controlled for these, the observational design cannot fully address residual confounding. Third, while the study was conducted across five centers in four European countries, the generalizability to other healthcare systems is uncertain. The small sample at center 2 (*n* = 10) limits the precision of that center’s estimates and its complete KPS adherence of 80% should be interpreted cautiously. Fourth, the operational definitions of some KPS components are complex, and the definitions used here may not be directly comparable to those used in other studies. While the definitions of the KPS components are based on the KDIGO guidelines and data from prior RCTs, specific thresholds can be challenged. Our MAP criterion required avoidance of MAP < 65 mmHg, but guidelines may suggest a range of thresholds. For example, the 2026 Surviving Sepsis Campaign guidelines [24] now suggest an initial MAP target of 60–65 mmHg for septic patients older than 65 years. Under our definitions, adherence to this updated recommendation would be classified as failed MAP optimization if MAP remained between 60 and 65 mmHg beyond the 12-hour grace period. This highlights that KPS operationalization involves threshold choices that may evolve with emerging evidence. Future consensus efforts should aim to harmonize KPS definitions reflecting current best evidence. Urine output monitoring showed the greatest between-center variation and represents the most actionable target for institutional quality improvement. However, the strict hourly documentation requirement may exceed what is necessary or feasible in routine practice, and many ICUs document urine output at longer intervals as part of routine practice. Future studies may evaluate whether less frequent, but regular, urine output monitoring intervals are equally effective in guiding clinical decision-making in AKI. Finally, risk for adverse outcomes among patients with AKI is not uniform. Clinicians may have selectively implemented KPS components in patients judged to be at higher risk. Improved risk prediction, using clinical variables or diagnostic tests might improve implementation.

## Conclusions

Among 258 critically ill adults with moderate or severe AKI enrolled across five European centers, the complete KDIGO-recommended Kidney Protective Strategy was fulfilled in less than one-third of patients. Successful implementation was associated with improved renal outcomes. Although exploratory, these findings identify actionable targets for ICU quality improvement and provide the rationale for future interventional trials evaluating structured nephroprotective strategy implementation.

## Supplementary Information

Below is the link to the electronic supplementary material.

Supplementary Material 1

## Data Availability

Due to ethical and legal restrictions (GDPR and consent limitations), potentially identifying data cannot be made publicly available. De-identified data are available upon reasonable request from the authors.

## Abbreviations

AKD: Acute Kidney Disease
AKI: Acute Kidney Injury
APACHE II: Acute Physiology and Chronic Health Evaluation II
BMI: Body Mass Index
CI: Confidence Interval
CKD: Chronic Kidney Disease
eGFR: Estimated Glomerular Filtration Rate
ICU: Intensive Care Unit
IQR: Interquartile Range
KDIGO: Kidney Disease: Improving Global Outcomes
KPS: Kidney Protection Strategy
MAP: Mean Arterial Pressure
RRT: Renal Replacement Therapy
SD: Standard Deviation
SHR: Subdistribution Hazard Ratio
SOFA: Sequential Organ Failure Assessment
STROBE: Strengthening the Reporting of Observational Studies in Epidemiology
