# Supplementary material for: Implementation of the kidney protection strategy in critically ill patients with acute kidney injury – a multi-center prospective cohort study
Source: Crit Care. 2026 Jun 19;30:319. doi: 10.1186/s13054-026-06144-0 (PMC13282845; doi:10.1186/s13054-026-06144-0)
Supplement: Supplementary file 1 — Supplementary Material 1 [file 13054_2026_6144_MOESM1_ESM.docx]

**Supplementary Table 1: Between-center variation in KPS component adherence.**

| **KPS Component** | **Center 1 (n = 47)** | **Center 2 (n = 10)** | **Center 3 (n = 25)** | **Center 4 (n = 142)** | **Center 5 (n = 34)** |
| --- | --- | --- | --- | --- | --- |
| Creatinine monitoring | 97.9 | 100.0 | 100.0 | 100.0 | 100.0 |
| Radiocontrast avoidance | 95.7 | 100.0 | 100.0 | 97.9 | 100.0 |
| Nephrotoxin management | 59.6 | 80.0 | 80.0 | 63.4 | 55.9 |
| Hemodynamic monitoring | 63.8 | 90.0 | 68.0 | 54.2 | 55.9 |
| Urine output monitoring | 97.9 | 90.0 | 28.0 | 61.3 | 35.3 |
| Hyperglycemia management | 42.6 | 80.0 | 32.0 | 42.3 | 47.1 |
| Optimization of MAP | 27.7 | 80.0 | 28.0 | 31.7 | 35.3 |
| **Complete KPS** | **25.5** | **80.0** | **28.0** | **28.9** | **35.3** |

**Supplementary Table 2: Distribution of the number of KPS components fulfilled.**

| **No. of bundle components fulfilled** | **N** | **Proportion** | **CI** |
| --- | --- | --- | --- |
| 0 | 0 | 0.000 | 0 – 0.014 |
| 1 | 0 | 0.000 | 0 – 0.014 |
| 2 | 32 | 0.124 | 0.086 – 0.171 |
| 3 | 59 | 0.229 | 0.179 – 0.285 |
| 4 | 58 | 0.225 | 0.175 – 0.281 |
| 5 | 22 | 0.085 | 0.054 – 0.126 |
| 6 | 7 | 0.027 | 0.011 – 0.055 |
| 7 | 80 | 0.310 | 0.254 – 0.37 |

**Supplementary Table 3: Exploratory analysis of individual KPS component effects.**

| **Component** | **SHR** | **CI low** | **CI high** | **P** |
| --- | --- | --- | --- | --- |
| **AKD beyond day 7** | | | | |
| Hemodynamic monitoring | 1.275 | 0.902 | 1.802 | 0.170 |
| MAP <65 mmHg | 1.497 | 0.985 | 2.277 | 0.059 |
| Nephrotoxic exposure | 1.242 | 0.876 | 1.761 | 0.220 |
| Urine output monitoring | 1.219 | 0.860 | 1.728 | 0.260 |
| Hyperglycemia management | 1.562 | 1.069 | 2.282 | 0.021 |
| Radiocontrast exposure | 1.851 | 0.885 | 3.870 | 0.100 |
| Creatinine monitoring | 3.060 | 2.567 | 3.646 | < 0.0001 |
| **Renal Recovery** | | | | |
| Hemodynamic monitoring | 0.935 | 0.594 | 1.471 | 0.770 |
| MAP <65 mmHg | 0.697 | 0.228 | 2.134 | 0.530 |
| Nephrotoxic exposure | 1.147 | 0.727 | 1.807 | 0.560 |
| Urine output monitoring | 0.648 | 0.417 | 1.006 | 0.053 |
| Hyperglycemia management | 0.789 | 0.470 | 1.324 | 0.370 |
| Radiocontrast exposure | 1.647 | 0.814 | 3.332 | 0.170 |
| Creatinine monitoring | 0 | 0.000 | 0.003 | <0.0001 |
| **RRT at day 30** | | | | |
| Hemodynamic monitoring | 5.377 | 1.836 | 15.751 | 0.002 |
| MAP <65 mmHg | 8.844 | 1.201 | 65.146 | 0.032 |
| Nephrotoxic exposure | 1.971 | 0.831 | 4.677 | 0.120 |
| Urine output monitoring | 0.766 | 0.301 | 1.949 | 0.580 |
| Hyperglycemia management | 1.662 | 0.652 | 4.236 | 0.290 |
| Radiocontrast exposure | 2.811 | 0.461 | 17.155 | 0.260 |
| Creatinine monitoring | 3.60 | 2.87 | 3.98 | < 0.0001 |

SHRs represent the effect of violation (non-adherence) vs. adherence (reference) for each component.

**Supplementary Table 4: Propensity score IPTW Fine–Gray models.**

| **Outcome** | **SHR** | **CI low** | **CI high** | **P** |
| --- | --- | --- | --- | --- |
| AKD (>7 days) | 0.624 | 0.365 | 1.068 | 0.086 |
| Renal Recovery | 5.502 | 3.276 | 9.240 | < 0.0001 |

RRT at 30 days: IPTW model could not be estimated due to complete separation (near-zero events in the weighted adherent group). No weighted estimate is reported.

# **Supplementary Table 5: Sensitivity analysis – Fine-Gray models adjusted for diabetes and hypertension.**

| **Outcome** | **SHR** | **CI low** | **CI high** | **P value** | **Adjusted for** |
| --- | --- | --- | --- | --- | --- |
| AKD | 0.71 | 0.42 | 1.20 | 0.19 | Age, AKI stage, SOFA Score, APACHE-II Score |
| AKD | 0.67 | 0.39 | 1.15 | 0.140 | Age, AKI stage, SOFA Score, APACHE-II Score, Diabetes, Hypertension |
| Renal Recovery | 6.29 | 3.08 | 12.85 | <0.001 | Age, AKI stage, CKD, SOFA Score, APACHE-II Score |
| Renal Recovery | 8.90 | 3.57 | 22.21 | <0.001 | Age, AKI stage, CKD, SOFA Score, APACHE-II Score, Diabetes, Hypertension |
| RRT at day 30 | 0.16 | 0.02 | 1.11 | 0.064 | Age, AKI stage, CKD, SOFA Score, APACHE-II Score |
| RRT at day 30 | 0.168 | 0.021 | 1.338 | 0.092 | Age, AKI stage, CKD, SOFA Score, APACHE-II Score, Diabetes, Hypertension |

**Supplementary Table 6: Clinical outcomes by KPS adherence**

| **Outcome** | **All Patients** | **KPS Fulfilled (n = 80)** | **KPS Not Fulfilled (n = 178)** | **p** |
| --- | --- | --- | --- | --- |
| **Renal outcomes** |  |  |  |  |
| Persistent AKI at 48 h — n/N (%) | 181/258 (70.2) | 57/80 (71.3) | 124/178 (69.7) | 0.80 |
| AKD beyond day 7 — n/N (%) | 152/258 (58.9) | 43/80 (53.8) | 109/178 (61.2) | 0.26 |
| Renal recovery at discharge — n/N (%)ᵃ | 90/155 (58.1) | 33/48 (68.8) | 57/107 (53.3) | 0.07 |
| RRT during ICU stay — n/N (%)ᵇ | 75/246 (30.5) | 27/77 (35.1) | 48/169 (28.4) | 0.30 |
| RRT at 30 days — n/N (%)ᶜ | 30/232 (12.9) | 2/73 (2.7) | 28/159 (17.6) | 0.002 |
| RRT at 90 days — n/N (%)ᵈ | 15/208 (7.2) | 2/65 (3.1) | 13/143 (9.1) | 0.13 |
| **ICU course** |  |  |  |  |
| ICU length of stay, days — median [IQR] | 11.7 [6.4–20.9] | 13.5 [7.5–23.1] | 10.8 [5.9–19.5] | 0.15 |
| **Mortality** |  |  |  |  |
| ICU mortality — n/N (%)ᵉ | 31/252 (12.3) | 12/78 (15.4) | 19/174 (10.9) | 0.31 |
| 30-day mortality — n/N (%)ᶜ | 38/232 (16.4) | 14/73 (19.2) | 24/159 (15.1) | 0.43 |
| 90-day mortality — n/N (%)ᵈ | 59/208 (28.4) | 20/65 (30.8) | 39/143 (27.3) | 0.61 |
| **MAKE** |  |  |  |  |
| MAKE30^f^ | 68 (29.3%) | 16 (21.9%) | 52 (32.7%) | 0.09 |
| MAKE90^f^ | 74 (35.6%) | 22 (33.8%) | 52 (36.4%) | 0.73 |

Crude unadjusted proportions; p-values from chi-square or Fisher's exact tests (categorical) or Wilcoxon rank-sum tests (continuous). These comparisons do not account for the competing risk of death. Competing-risk regression results are reported in Table 3. ᵃ Among hospital survivors with available baseline and discharge renal function data. ᵇ Among patients with evaluable ICU RRT data (n = 246). ᶜ Among patients with evaluable 30-day follow-up data. ᵈ Among patients with evaluable 90-day follow-up data. ᵉ Among patients with evaluable ICU discharge data. ^f^ Due to the unavailability of post-discharge creatinine/eGFR, MAKE is defined as death or dialysis.


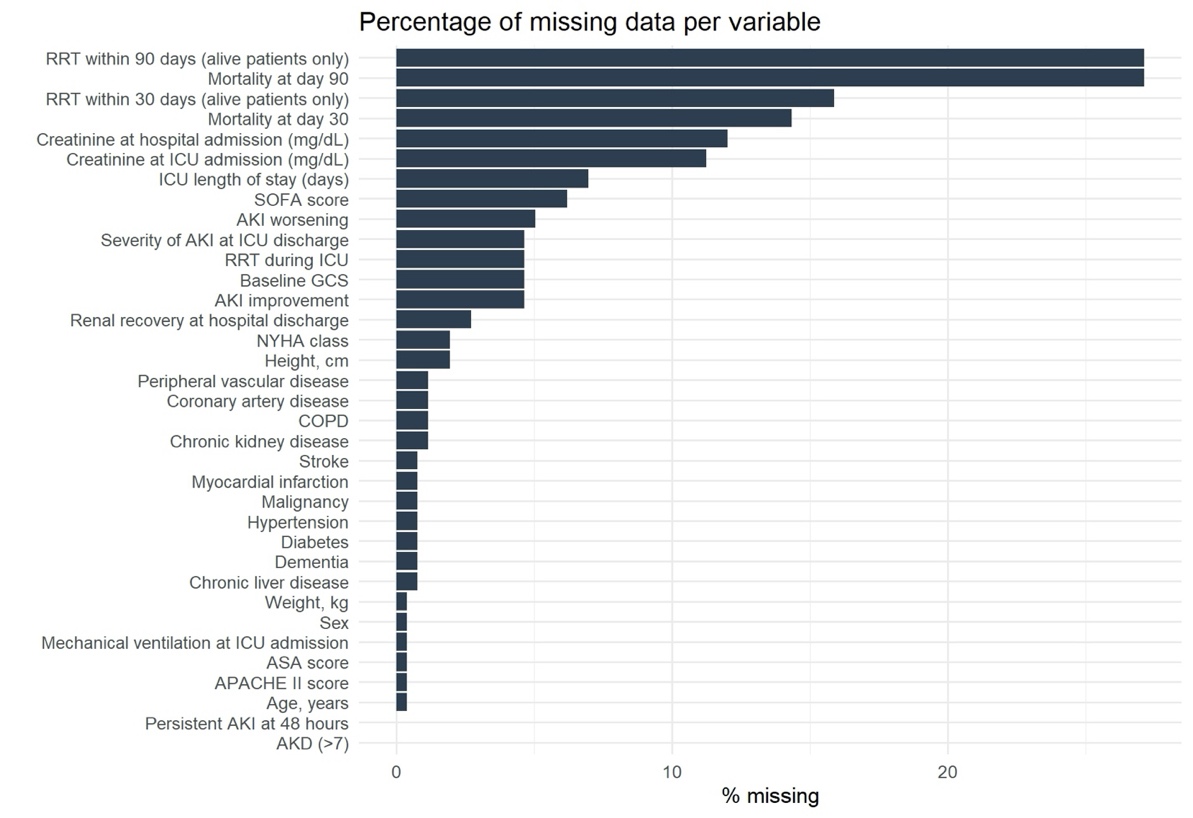


**Supplementary Figure 1: Missing Data.**


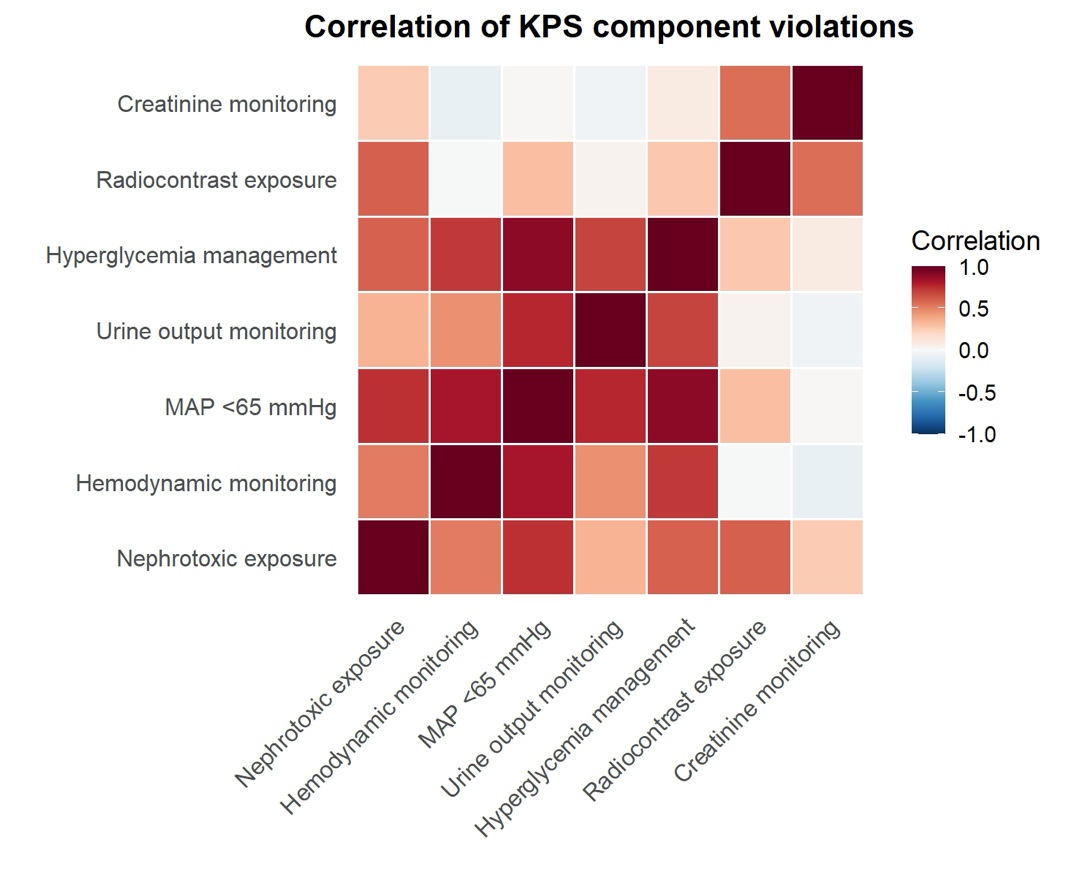


**Supplementary Figure 2: Correlation of KPS component violations.** In the correlation analysis of KPS component violations, distinct patterns of co‑occurrence emerged. Violations clustered primarily within two domains. A **hemodynamic cluster** – including hemodynamic monitoring, MAP < 65 mmHg – showed strong positive correlations, indicating that patients failing one of these monitoring or optimization steps often failed the others. A second **exposure cluster** – nephrotoxic exposure and radiocontrast exposure – displayed moderate inter‑correlations, suggesting that lapses in renal protection and monitoring tended to co‑occur. Hyperglycemia management correlated modestly with both clusters.


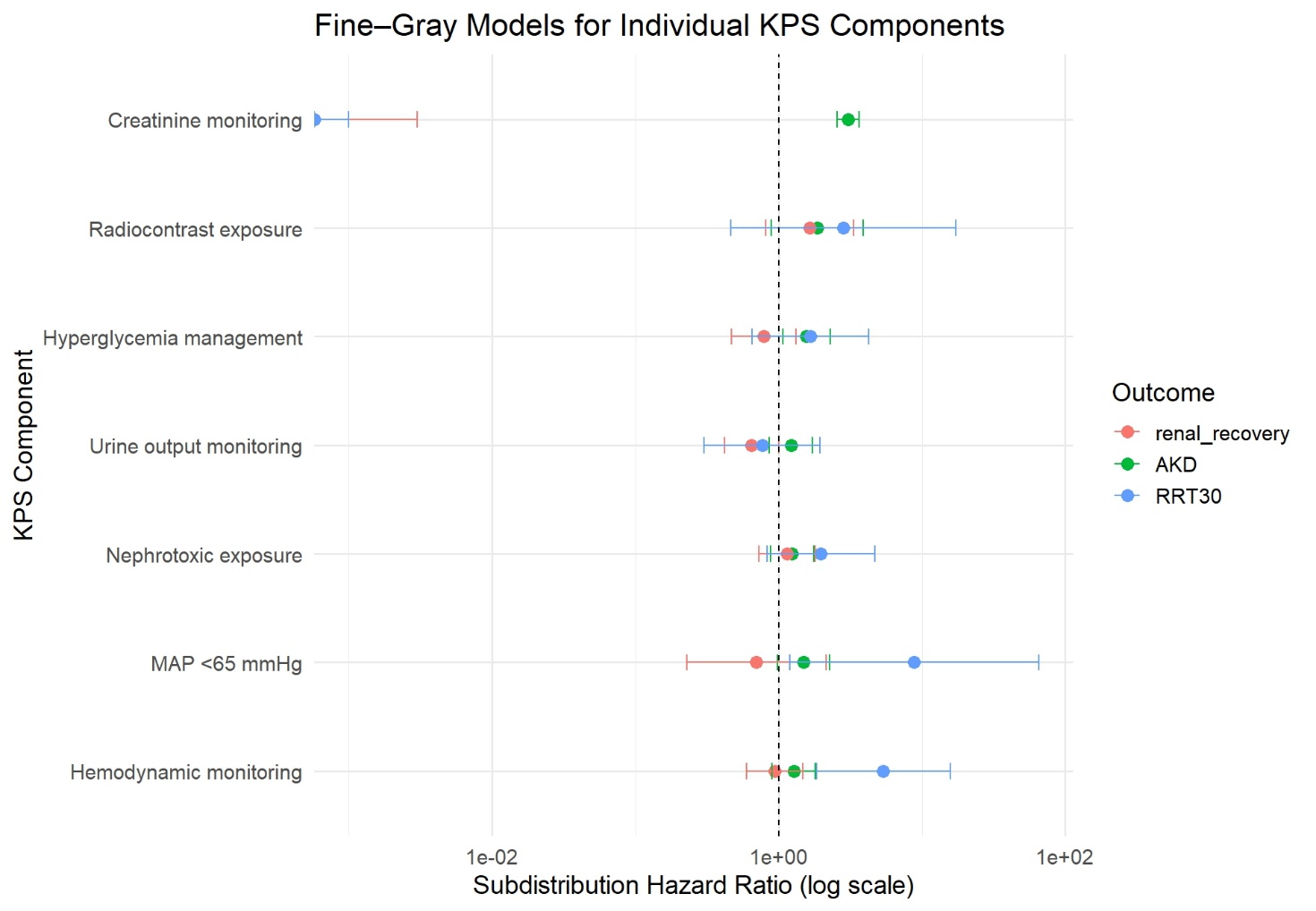


**Supplementary Figure 3: Individual component effects.** Forest plot showing subdistribution hazard ratios (log scale) for each individual KPS component violation across three renal outcomes: AKD beyond day 7 (green), renal recovery at discharge (red), and RRT at 30 days (blue). Each point represents the estimated SHR with 95% confidence intervals from univariable Fine–Gray competing risk models. The vertical dashed line indicates the reference value (SHR = 1). Violations of MAP < 65 mmHg and hemodynamic monitoring were most strongly associated with adverse renal outcomes while the strong apparent association with creatinine should be interpreted as ascertainment bias as creatinine measurements are required for outcome documentation.


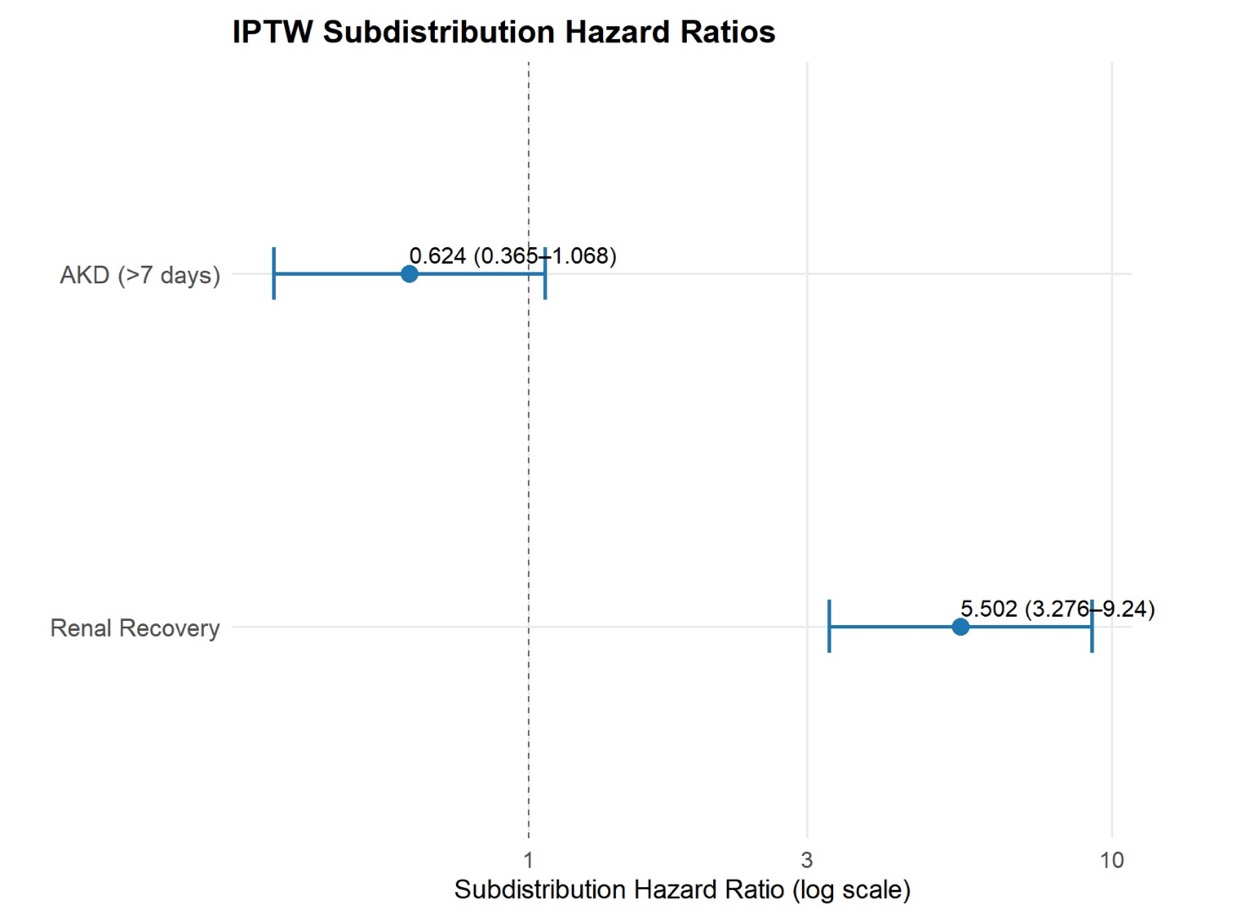


**Supplementary Figure 4: IPTW Hazard ratios.** IPTW analysis confirmed the strong association between KPS fulfillment and renal recovery (SHR 5.50; 95% CI 3.28–9.24; p < 0.0001). For AKD, the association showed a consistent trend (SHR 0.62; 95% CI 0.37–1.07; p = 0.086). RRT at 30 days: IPTW model could not be estimated due to complete separation (zero/near-zero events in the weighted adherent group). No weighted estimate is reported
